# Effect of mouse nerve growth factor on the expression of glial fibrillary acidic protein in hippocampus of neonatal rats with hypoxic-ischemic brain damage

**DOI:** 10.3892/etm.2012.827

**Published:** 2012-11-23

**Authors:** XIAOJUAN YIN, LEI DONG, WEI WEI, YU WANG, YANNAN CHAI, ZHICHUN FENG

**Affiliations:** 1 Affiliated Bayi Children’s Hospital, Beijing Military Region General Hospital, Beijing 100700;; 2 Clinical Laboratory Center, PLA Air Force General Hospital, Haidian District, Beijing 100142, P.R. China

**Keywords:** neonatal rat, hypoxic-ischemic brain damage, mouse nerve growth factor, glial fibrillary acidic protein

## Abstract

The present study aimed to investigate the influence of mouse nerve growth factor (mNGF) on glial fibrillary acidic protein (GFAP) expression in neonatal rats with hypoxic-ischemic brain damage (HIBD). A total of 60 7-day-old neonatal rats were randomly divided into control, HIBD and mNGF groups (n=20). The rats in the mNGF group were injected intramuscularly with mNGF once a day for 5 days. Each group was randomly divided into a day 7 subgroup and a day 14 subgroup according to the time of sacrifice. After the rats were sacrificed, the expression of GFAP in the hippocampus in the three groups was confirmed by immunohistochemical analysis. The results revealed that the expression level of GFAP in the ischemic side of the hippocampus in the mNGF and HIBD groups was higher compared with that in the control group at days 7 and 14 after surgery, respectively (P<0.01). GFAP-positive cells were mainly distributed in the ischemic side of the hippocampal dentate gyrus (DG) region in the mNGF group while in the HIBD group they were in the ischemic side of the hippocampal CA1 region. Compared with day 7, the expression of GFAP in the ischemic side of the hippocampus in the mNGF group increased at 14 days (P<0.01), but decreased in the HIBD group (P<0.01); however, this was still higher than that in the control group (P<0.01). This study revealed that mNGF increases the expression of GFAP in the hippocampus of neonatal rats with HIBD and therefore may have a role in the repair of HIBD.

## Introduction

Hypoxic-ischemic brain damage (HIBD), a result of asphyxia in the perinatal period, seriously threatens the health and life of newborns. HIBD leads to apoptosis and necrosis of nerve cells, with a high mortality rate and poor outcome. Long-term nervous system sequelae, including disorders of recognition, language, sensation, movement, vision and memory function affect 25–30% of patients with HIBD. To date, there is no specific remedy for HIBD (1–4). Earlier interruption of the pathological and physiological changes of HIBD is the key point for decreasing case fatality and disability rate of newborn infants. In spite of the great progresses that have been made in treatment, the therapeutic efficacies remain unsatisfactory. Therefore, the absence of effective therapies for HIBD has provoked an intensive search for novel treatment strategies. A previous study demonstrated that mNGF may have a potential therapeutic efficacy for HIBD; however, little is known about its mechanism (5).

Glial fibrillary acidic protein (GFAP), a skeleton protein generated from astrocytes, is crucial in the survival of neurons and formation of synapses following brain injury (6). In this study, using a neonatal mouse model of HIBD, we investigated the expression of GFAP in neonatal rats with HIBD when injected with mouse nerve growth factor (mNGF) and determined whether mNGF promotes the accrementition of astrocytes. This study provides experimental evidence for the treatment of HIBD and correlated diseases with mNGF.

## Materials and methods

### Animals, grouping and intervention measures

A total of 60 Sprague-Dawley (SD) neonatal rats aged 7 days and weighing 10–12 g (specific pathogen-free) were purchased from the Center for Laboratory Animals of Academy of Military Medical Sciences (Beijing, China). The rats were randomly assigned into 3 groups: control, HIBD and mNGF groups (n=20 per group). According to the time of sacrifice, each group was divided into 2 subgroups (days 7 and 14). In the mNGF group, neonatal rats with HIBD were injected intramuscularly into the buttocks with 20 ng/g/day mNGF for 5 days. In the HIBD group, neonatal rats with HIBD received no treatment. In the control group, neonatal rats were intramuscularly injected with 10 μl/g/day 0.1 M PBS solution for 5 days. On days 7 and 14 post-treatment, animals were sacrificed for further experiments (n=10 per time point).

### Main reagents

Rabbit anti-rat GFAP polyclonal antibody was purchased from Chemicon (Temecula, CA, USA). A 3,3′-diaminobenzidine (DAB) visualization kit, tetrazolium red, cyanine-3 (cy3) and a streptavidin-biotin complex (SABC) kit were purchased from Zhongshan Biotech Co. (Beijing, China) mNGF was purchased from Wuhan Haite Biological Pharmaceutical Co. (Wuhan, China). Other domestic reagents (analytically pure) were used in this study.

### Establishment of the HIBD rat model

The neonatal HIBD model was established as previously described (7–9). The 7-day-old SD rats were anesthetized with anhydrous diethyl ether for 0.5–1 min. Then, the animals were placed in the left palm under a microscope. The forefinger and middle finger were used to fix the head of the animal and the thumb to fix the bilateral forelimbs. A midline incision was made at the neck and the subcutaneous fat was separated. The left carotid artery was isolated and ligated with 5-0 double suture. Hemostasis was performed using a gelatin sponge. Following the procedures, the rats were placed into a transparent plastic container, placed in a 37°C water bath and ventilated with a constant flow of a humidified mixture of 8% oxygen and 92% nitrogen for 2.5 h. Once the HIBD rat model was established, the rats in the mNGF group were injected intramuscularly with mNGF.

### Sample collection

At the different time points, 3 groups of rats were sacrificed under anesthesia via inhalation of anhydrous diethyl ether. The heart and aorta were exposed. Blood in the circulation was removed by perfusion with 20 ml/kg cold normal saline via an aortic cannula. The brain was collected, the macroscopic features of which were recorded and was placed in a freezing microtome at −200°C for 2 h. Frozen tissue sections (8 μm) were placed on slides previously treated with polylysine, fixed with acetone for 20 min and stored at −20°C on standby.

### Tetrazolium red staining

One rat was sacrificed in each group and the brain was collected and stained with 2% tetrazolium red. The white region was defined as the ischemic area.

### Detection of GFAP expression in the hippocampus

The SABC immunohistochemical analysis was performed to determine the GFAP expression in the hippocampus. After being placed at room temperature for 20 min, the tissue sections were treated as follows: treated with dimethyl benzene and a series of increasing ethanol concentrations for deparaffinization and dehydration; incubated with 0.6% methanol in H_2_O_2_ for 20 min; washed in phosphate-buffered saline (PBS) three times (5 min each); blocked with goat serum at room temperature for 20 min; incubated at 37°C with rabbit anti-rat GFAP polyclonal antibody (1:100) for 90 min; washed in PBS three times (10 min each); incubated at 37°C with biotinylated goat anti-rabbit IgG antibody for 30min; washed in PBS three times (10 min each); treated with SABC at 37°C for 30 min; washed in PBS four times (5 min each) and visualized with DAB for 5 min. Then the sections were observed under a light microscope. During lab processing, if the biotinylated goat anti-rabbit IgG antibody was replaced by goat anti-rabbit IgG antibody marked with cy3, the sections were observed under a fluorescent microscope after incubation for 30 min at 37°C with SABC and three washes with PBS (10 min each). In the negative control group, the primary antibody (rabbit anti-rat GFAP polyclonal antibody) and the secondary antibody (biotinylated goat anti-rabbit IgG antibody) were replaced with 0.01 mol/l PBS and goat serum, respectively. Positive cells with GFAP expression in tissues colorated with DAB or cy3 were bown-yellow or red, respectively. Positive cells were counted at a magnification of ×200. Three sections were selected from each sample and 4 visual fields were randomly selected from each section. Positive cells in 12 visual fields were counted in each sample and the number of GFAP-expressing cells was determined in each subgroup.

### Statistical analysis

Statistical analysis was performed with SPSS version 12.0 (SPSS Inc., Chicago, IL, USA). All data were shown as mean ± standard deviation (SD) for normally distributed continuous variables. One way analysis of variance was performed to determine comparisons among different groups. P<0.05 was considered to indicate a statistically significant difference.

## Results

### Behavior of rats with HIBD

After ligation of the left common carotid artery, the following occurred at different hypoxic durations: hypoxia for 10 min induced dysphoria; hypoxia for 15–20 min caused cyanosis and deep and rapid breathing; hypoxia for 20–30 min led to unstable standing and a dragging step of the right hind limb during creeping; hypoxia for 35–60 min significantly reduced the activity and hypoxia for more than 1 h resulted in lethargy and irritability in 90% of rats with HIBD. At 1 h after post-hypoxic re-oxygenation, rats circled towards the left side. Abnormal behavior was not observed in the rats receiving hypoxia alone.

### Tetrazolium red staining

In the HIBD group and mNGF group, the ischemic and intact hemispheres were white and red, respectively.

### Morphological characteristics of GFAP-expressing cells

**Figure 1.**
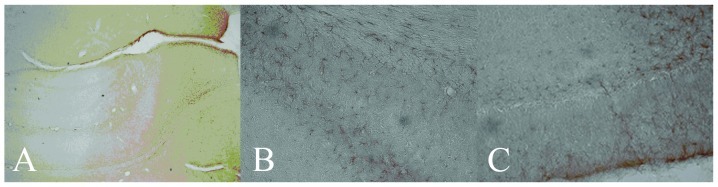
GFAP protein expression in the hippocampus of different groups at day 7 (DAB staining; positive cells, brown-yellow). (A) Control group (hippocampus; magnification, ×100); (B) HIBD group (CA1; magnification, ×200) and (C) mNGF group (DG; magnification, ×200). GFAP, glial fibrillary acidic protein; DAB, 3,3′-diaminobenzidine; HIBD, hypoxic-ischemic brain damage; mNGF, mouse nerve growth factor; DG, dentate gyrus.

**Figure 2.**
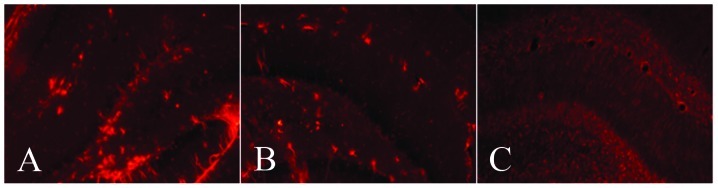
GFAP protein expression in the hippocampus of different groups at day 14 (cy3 staining; positive cells, red). (A) control group; (B) HIBD group and (C) mNGF group (hippocampus; magnification ×100). GFAP, glial fibrillary acidic protein; cy3, cyanine-3; HIBD, hypoxic-ischemic brain damage; mNGF, mouse nerve growth factor.

The GFAP protein was mainly found in the cytoplasm, stained brown-yellow by DAB or red by cy3, respectively. The expression levels of GFAP in the ischemic side of the hippocampus in the mNGF and HIBD groups were higher than those in the control group at days 7 and 14 after the intervention. GFAP-positive cells in the mNGF and HIBD groups were mainly distributed in the ischemic side of the hippocampal dentate gyrus (DG) and CA1 region, respectively (Figs. 1 and 2).

### Comparison of GFAP-expressing cells in the hippocampus among different groups

The number of GFAP-expressing cells and the extent of staining were different in the 3 groups at day 7 (F=58.94, P<0.01) and day 14 (F=79.57, P<0.01). The number of GFAP-expressing cells in the mNGF group was higher than that in the HIBD and control groups. In the HIBD group, the number of GFAP-expressing cells was significantly increased when compared with that of the controls. Compared with day 7, the number of GFAP-expressing cells in the mNGF group significantly increased at day 14 (P<0.01), but decreased in the HIBD group; however, this was still lower than that in the mNGF group and higher than that in the control group (P<0.01; Table I).

## Discussion

Astrocytes, the most common cell type in the central nervous system, play an important role in the association of neurons and cerebral vessels, meninges and ventricles of the brain (10). GFAP, a specific protein expressed by mature astrocytes, is an important marker of colloid cellular proliferation following a central lesion, which promotes cariocinesis of astrocytes and differentiation from primitive ancestral cells to mature astrocytes (11).

Newborn HIBD is a disease where hypoxia, ischemia and a decrease in cerebral blood flow occur as a result of various factors in the perinatal period. The hippocampus of newborns is fragile and injures easily under exposure to an hypoxicischemic condition. The amount of colloid cellular apoptosis is the maximum total count of nerve cell death (12). In addition, effective treatment for newborn hypoxic-ischemic brain damage is limited. To date, although little is known about the mechanism of nerve injury and regeneration, neurotrophic factors have been considered to be of potential value for HIBD therapy. In this study, GFAP-expressing cells increased compared with those of the control group following cerebral anoxia and ischemia, which indicates that GFAP may be involved in the recovery of HIBD.

NGF, the first type of typical neurotrophic factor to be described and studied, exists commonly in tissues of animals. It promotes activation of impaired nerves and has a close association with the development, functional maintenance and reparation of the nervous system (13). NGF, as a nutrition factor of the central nervous system, facilitates regeneration of impaired neurons for improvement of the pathological state and has the ability to protect and repair nerves impaired by hypoxia and ischemia. By enhancing the dendritic process generation and expansion and survival of nerve cells, NGF increases the density of nerve fibers in a dominant target region, particularly in the hippocampus, basal forebrain and cholinergic neurons of the corpora striata. NGF not only promotes the growth and maintains the survival of sympathetic and sensory neurites, but also enhances the karyokinesis and differentiation of nerve cells following an increase in nerve cells. In addition, NGF determines the growth direction of axons (14,15). Studies have suggested that upregulation of NGF expression has a positive effect on the protection of impaired neurons. The activities of free radical scavengers, including hydrogen peroxidase, superoxide dismutase and glutathion peroxidase, are intensified by NGF, which lessens the degree of injury of nerve cells; neurons may be protected by the rivalry of the neurotoxicity of excitatory amino acids and the regulation of calcium ion levels in neural intracytoplasm by NGF and the degree of nerve cell injury may be relieved by NGF through inhibition of programmed cell death and increased cerebral blood flow (16). Nakatomi *et al*(17) suggested that the infusion of NGF to cerebral ventricles following cerebral ischemia significantly enhances the activation and proliferation of neural stem cells (NSCs) and improves the symptoms of neurological impairment. mNGF is a major molecule of the NGF family. A study has suggested that mNGF has a high therapeutic efficacy for HIBD patients; however, the mechanism of action remains unknown (5).

In this study, the number of GFAP-expressing cells increased following cerebral anoxia and ischemia; however, the number of GFAP-expressing cells decreased with an extended anoxia-ischemia time. mNGF promotes the expression of GFAP in the hippocampus of HIBD. In addition, there was no trend in the downregulation of GFAP expression with extending anoxia-ischemic time. In conclusion, we confirm the potential of mNGF in a repairing role for HIBD, which indicates that mNGF may be involved in the recovery of HIBD by regulating the glial cell response and enhancing the accrementition of astrocytes.

## Figures and Tables

**Table I.** Number of cells positive for GFAP in the hippocampus of different groups.

Group	Day 7	Day 14	P-value
Control	19.01±3.75	20.50±1.12	0.0968
HIBD	43.52±3.05^a^	25.53±1.09^a^	<0.01
mNGF	57.32±3.98^a,b^	62.78±1.22^a,b^	<0.01
F-value	58.94	79.57	
P-value	<0.01	<0.01	

a Significantly different from the control group by Tukey’s post hoc test (P<0.05);

b significantly different from the HIBD group by Tukey’s post hoc test (P<0.05). Data represent mean ± standard deviation. GFAP, glial fibrillary acidic protein; HIBD, hypoxic-ischemic brain damage; mNGF, mouse nerve growth factor.
